# Unveiling diverse interactions between soil microbiota and host plants for sustainable agriculture: current limitations and prospects

**DOI:** 10.3389/fmicb.2026.1841614

**Published:** 2026-08-26

**Authors:** Moazma Batool, Sadam Hussain, Abdul Ghaffar Shar, Hafeez ur Rehman, Ejaz Ahmad Waraich, Arslan Haidar, Usman Zulfiqar, Ruixin Shao, Jwaher Salem Khamis Alghafri, Mayank Anand Gururani

**Affiliations:** 1 Department of Botany, University of Agriculture, Faisalabad, Pakistan; 2 College of Agronomy, Henan Agricultural University, Zhengzhou, China; 3 College of Grassland Agriculture, Northwest A&F University, Yangling, China; 4 Xi’an Fanyi University, Taiyigong, Shaanxi, China; 5 Department of Agronomy, University of Agriculture, Faisalabad, Pakistan; 6 Department of Agronomy, Faculty of Agriculture and Environment, The Islamia University of Bahawalpur, Bahawalpur, Pakistan; 7 Department of Biology, Nakhchivan State University, Nakhchivan, Azerbaijan; 8 Key Laboratory of High-Efficiency Production of Wheat-Maize Double Cropping/College of Agronomy, Henan Agricultural University, Zhengzhou, China; 9 Department of Biology, College of Science, United Arab Emirates University, Al Ain, United Arab Emirates

**Keywords:** microbial communication, signaling, soil microbes-interaction, soil properties, sustainable agriculture

## Abstract

The intricate relationship between soil microbiota and host plants plays a pivotal role in maintaining soil health and sustaining agricultural productivity. In this review, we examined current knowledge of these interactions, highlighting both their significance and the limitations in existing research. Although substantial progress has been made in elucidating the roles of diverse microbial communities in nutrient cycling, plant growth promotion, and disease suppression, several challenges remain. These include the complexity and diversity of microbial communities, as well as the dynamic nature of soil–plant interactions under varying environmental conditions. Furthermore, there is a need for greater integration of interdisciplinary approaches, encompassing molecular biology, microbiology, ecology, and agronomy, to effectively address these challenges. In this context, this study proposes future research directions aimed at advancing our understanding of soil microbiota-plant interactions and their implications for sustainable agriculture. These include the development and application of advanced omics techniques, such as metagenomics and metatranscriptomics, to comprehensively characterize microbial communities and their functional attributes. Furthermore, harnessing the potential of microbial inoculants and biofertilizers tailored to specific crops, soils, and environmental conditions represents a promising strategy for improving soil health, enhancing nutrient use efficiency, and ensuring sustainable crop production. Overall, addressing these research gaps and leveraging emerging technologies will deepen our understanding of soil microbiota-plant interactions and facilitate the development of innovative, science-based strategies to promote resilient and sustainable agricultural systems.

## 1 Introduction

Soil microorganisms, such as bacteria, actinomycetes, viruses, fungi, nematodes, and protozoa, represent one of the largest reservoirs of biodiversity and play essential roles in a wide range of biological processes in terrestrial ecosystems (FAO, ITPS, GSBI, SCBD, and EC, 2020; Cheng et al., 2026). Ecosystem multifunctionality refers to the simultaneous provision of multiple ecosystem functions and services, which are largely determined by the ecological activities of organisms inhabiting a given environment (Slade et al., 2017). Soil microorganisms mediate the biogeochemical cycles of carbon (C), nitrogen (N), sulphur (S), and phosphorus (P), while also playing a fundamental role in the decomposition of organic matter. Through these processes, they contribute significantly to soil health and ecosystem stability. These microbes facilitate biological N fixation and various biological transformations that enhance nutrient availability, promote seedling growth, and improve soil quality. They also increase the availability of essential nutrients, thereby supporting crop productivity and nutrient recycling within soil ecosystems. In particular, soil microbes are crucial for N cycling and organic matter decomposition (Shah et al., 2021). Overall, microbial communities are indispensable for maintaining terrestrial ecosystem stability because they regulate numerous soil processes, including biogeochemical cycling, plant productivity, and climate regulation.

Soil is a valuable but largely nonrenewable resource that supports immense microbial diversity (Sharma et al., 2024). Soil microbial populations play substantial roles in the degradation of various compounds, including organic contaminants such as insecticides (Barra Caracciolo and Grenni, 2021). In addition, they promote plant growth and contribute to disease control (Saleem et al., 2019). The biomass, diversity, and activity of soil microorganisms are major drivers of ecosystem processes that sustain agricultural productivity. A diverse and active microbial community is widely recognized as an indicator of healthy soil and is essential for optimal plant growth and crop yield (Paz and Fu, 2016). Moreover, soil microorganisms enhance plant resistance to diseases by inducing systemic resistance and forming protective barriers around root surfaces that limit pathogen invasion. By increasing plant resistance to biotic stresses, these microbes provide valuable opportunities for sustainable food production. One notable example is biological pest control, which utilizes beneficial microorganisms to manage agricultural pests. The bacterium *Bacillus thuringiensis* (Bt), for instance, is widely produced and applied as a biological agent against caterpillars and other insect pests (University of York, 2019).

The composition and diversity of soil microbial communities are strongly influenced by abiotic factors. Although the ecological mechanisms governing soil microbial systems are not yet fully explored, microorganisms are estimated to mediate approximately 80–90% of soil processes (Sokol et al., 2022). These organisms participate in complex ecological interactions that influence soil structure, nutrient cycling, organic matter decomposition, mineral nutrient release, and plant performance. Despite their recognized importance, the mechanisms underlying many of these interactions remain poorly understood. Nevertheless, the beneficial functions of soil microbiota are largely dependent on their synergistic associations with other soil organisms and environmental components.

Microbial activity can significantly influence the physical, chemical, and biological characteristics of soil. According to an estimate, 1 g of soil contains between 10^8^ and 10^10^ microbial cells (Zhang et al., 2017). These highly complex communities, comprising hundreds to thousands of bacterial, fungal, viral, and archaeal species, vary considerably among soil types and environmental conditions (Wu et al., 2018). Agricultural intensification, for example, can disrupt the ecological balance required to maintain soil health, often resulting in reduced microbial diversity compared with natural or undisturbed soils (Zhou et al., 2024).

Numerous biotic and abiotic factors influence microbial populations within the rhizosphere. The structure, composition, and dynamics of belowground microbial communities are governed by multiple ecological mechanisms, including plant-microbe interactions and rhizosphere-mediated effects (Singh et al., 2024). Certain beneficial bacteria can colonize plants during the early stages of root development and may even be vertically transmitted through seeds to subsequent plant generations (Malinich and Bauer, 2018). In addition, plant host identity and specific plant compartments significantly influence the composition of associated microbial communities, both above and below ground (Zeng et al., 2024).

Although substantial progress has been made in the study of beneficial microorganisms for crop production, the development of effective field-scale, bio-based plant protection strategies remains limited. This highlights the significance of understanding the survival, establishment, and functional roles of introduced microorganisms within agroecosystems, as well as the ecological significance of crop-associated microbiomes. Soil harbors vast populations of microorganisms that decompose organic matter, enhance soil fertility, and support sustainable agricultural production (Toor and Adnan, 2020). This review examines the diversity and ecological significance of soil microbes and their roles in maintaining soil fertility, supporting plant growth, and promoting sustainable agriculture. Furthermore, it discusses current limitations, challenges, and future prospects in understanding soil-microbe interactions and their application in agricultural systems.

## 2 Interactions between soil microbes and host plants

Plants have the ability to establish connections with a diverse range of microorganisms present in the soil. These interactions may be positive, neutral, or harmful. The outcome of plant-microorganism interactions is determined by various factors such as the plant’s growth stage, soil composition and structure, and the presence of specific microbial communities (Monaco et al., 2024). These microbial communities are collectively referred to as the plant microbiome. Numerous microbial species react to the rhizosphere environment, which is influenced by root exudates, to form this microbiome. The diversity of these organisms determines the nature of plant interactions and the complex relationships within the plant holobiont (Table 1; Etalo et al., 2018).

**Table 1:** Soil microbes’ groups and their functions in plants.

Microbial group	Function	Examples	References
*Plant Growth-Promoting Rhizobacteria*	Play a role in the synthesis of ACC de-amines and phosphorus solubilization. Enhanced expression of systemic tolerance genes and increased root volume.	*Rhizobium* spp. *Azotobacter* and *Acetobactor*	Xu et al. (2018)
Fungal endophytes	Enhanced tolerance to abiotic and biotic stresses, promoted plant growth through modulation of phytohormones, increased nutrient uptake and transport.	Epichloe species, facultative biotrophic fungi *and Piriformospora indica*	Li et al. (2018)
Bacteria	Increased nitrogen fixation and improved plant growth	*Rhizobium* spp.	Menge et al. (2019)
Fungi	Improved nutrient and water uptake and promoted plant growth	Mycorrhizal fungi	Smith and Read (2008)
Protozoa	Regulated bacteria populations and nutrient cycling	Amoebae, flagellates, and ciliates	Bonkowski (2004)
Algae	Enhanced nitrogen fixation and soil stabilization	Cyanobacteria (blue-green algae)	Whitton and Potts (2007)

### 2.1 Rhizobium

Rhizobium is a symbiotic bacterium belonging to the family Rhizobiaceae. It forms mutualistic associations with legumes and certain non-legumes like *Parasponia*, enabling the fixation of atmospheric nitrogen. This association benefits forage legumes such as lucerne and berseem, as well as pulse crops like chickpea, pigeon pea (redgram), and pea. The bacteria invade plant roots and form specialized structures called nodules, which function as sites for ammonia synthesis (Saifi et al., 2024). These nodules contain differentiated bacteroids that convert atmospheric nitrogen into ammonia, serving as a readily available nitrogen source for crop plants. In return, the bacteria receive a specific habitat and nutrients from the host plant (Das et al., 2022).

### 2.2 Azotobacter

Azotobacter is a free-living, heterotrophic bacterium belonging to the family Azotobacteriaceae. Although it thrives in low oxygen environments, it is an obligate aerobe (Himabindu and Goutami, 2024). It is capable of fixing atmospheric nitrogen independently. Reported species include *A. macrocytogenes*, *A. beijerinckii*, *A. insignis*, and *A. vinelandii*. Populations of Azotobacter are typically low in uncultivated soils but are commonly found in the rhizosphere of crops such as rice, maize, sugarcane, millet, vegetables, and plantation crops.

Azotobacter plays a significant role in the nitrogen cycle by converting atmospheric N into ammonium, a form easily available to plants (Chamoli et al., 2024). It possesses key enzymes required for nitrogen fixation, including nitrogenase, ferredoxin, and hydrogenase (Zeng et al., 2024). This process requires energy in the form of adenosine triphosphate (ATP) (Rajesha and Ray, 2020). Since nitrogenase is highly sensitive to oxygen, Azotobacter has evolved defense mechanisms, such as increased respiratory activity to reduce the oxygen level in the cell (Negi et al., 2024). Additionally, protective proteins such as Shethna protein help protect nitrogenase from oxygen damage. Some Azotobacter species also produce phytohormones like auxins, which promote plant growth (Kumar et al., 2024).

### 2.3 Mycorrhizae

Mycorrhizae refer to the mutualistic symbiotic interaction between soil-borne fungi and plant roots (Singh et al., 2019). In this relationship, fungal hyphae extend beyond the root zone, increasing the plant’s ability to absorb available resources. These fungi produce enzymes that facilitate the breakdown of soil organic matter, releasing phosphorus and nitrogen, and also secrete organic acids that help mineral weathering. In return, plants supply the fungi with carbohydrates that are important for their growth and metabolism. It is estimated that 4–20% of a plant’s net photosynthates may be allocated to its fungal companions (Gorka et al., 2019).

Mycorrhizal associations play a central role in P mobilization, particularly in acidic tropical soils (Seguel et al., 2013). Arbuscular mycorrhizal fungi (AMF), in particular, are important for P cycling, mobilization, and uptake in such environments (Klugh and Cumming, 2007). These fungi form an extensive hyphal network that acts as extensions of the root system, exchanging absorbed *p* for plant-derived carbon (Xu et al., 2007; Zhang et al., 2016).

Additionally, the hyphosphere - the area surrounding fungal hyphae – serves as a hotspot for microbial activity. AMF can facilitate the transfer of recently fixed C to soil bacteria (Zhang et al., 2014; Manchanda et al., 2017). The associated AMF microbiota contributes to numerous plant growth-promoting processes, including phytate mineralization, siderophore production, phosphate solubilization, and the formation of low-molecular-weight organic acids (Battini et al., 2016).

In contrast, negative associations between plants and microorganisms are classified as parasitism or predation (Ngah et al., 2018). Pathogenic microbes exploit plant resources such as nutrients and water, adversely affecting plant growth, development, and overall health (Li et al., 2024).

**Figure 1:**
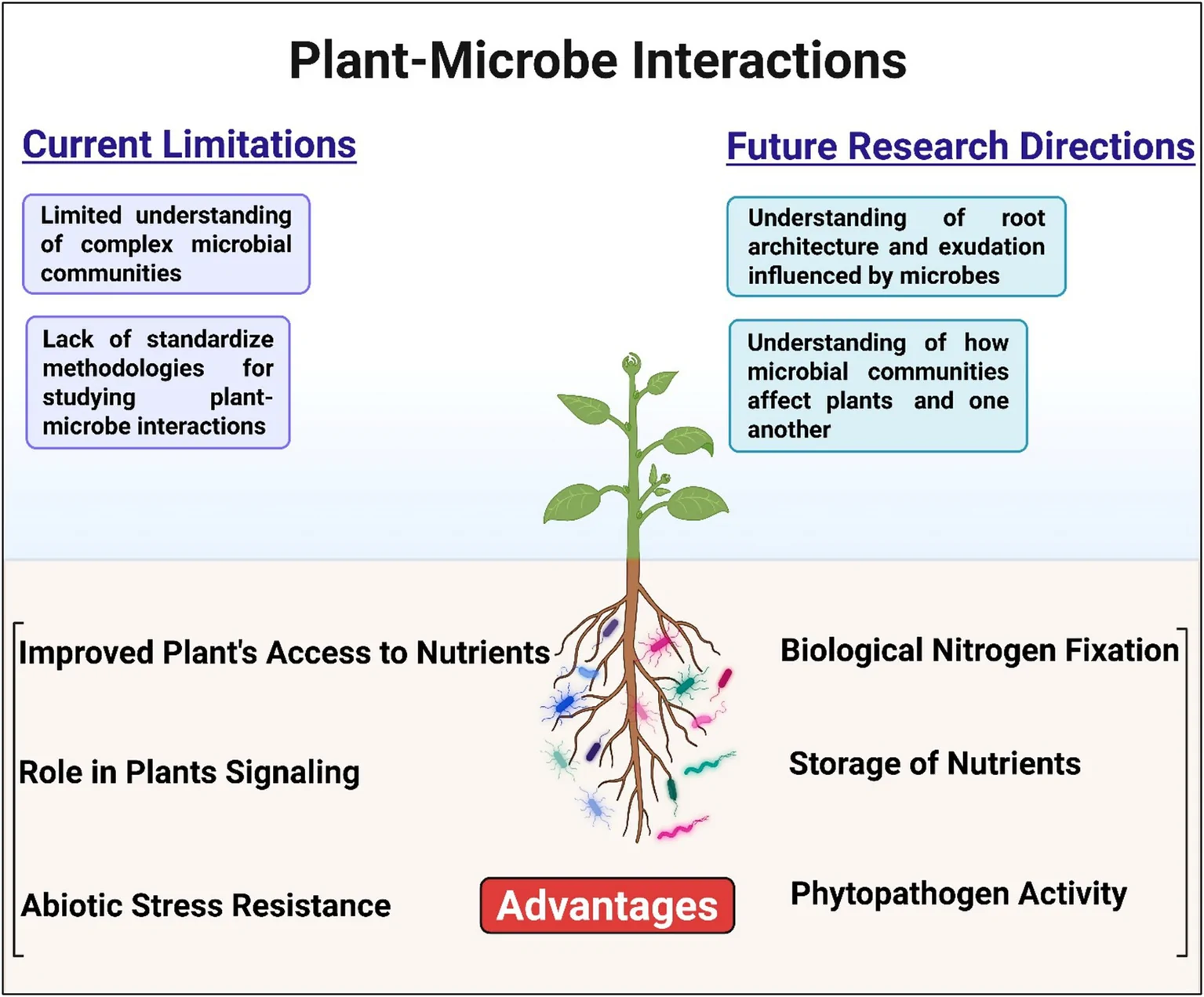
Graphical abstract for interactions between soil microbiota and host plants for sustainable agriculture.

According to an estimate, most terrestrial plants form mutualistic relationships with AMF, a dominating group of soil fungi. In this association, plants receive essential nutrients such as phosphorus in exchange for carbon. Because AMF are obligate symbionts, they cannot complete their life cycle independently and persist in soil as spores until they encounter a suitable host (Tkacz and Poole, 2015). Upon germination, hyphae grow toward plant roots, guided by root exudates such as strigolactone. The fungi then form specialized structures called appressoria to penetrate root tissues, mediated by signaling molecules such as lipochitooligosaccharides (Maillet et al., 2011). Ultimately, highly branched hyphal structures develop within cortical cells, surrounded by the plant plasma membrane, supporting nutrient exchange between the symbiotic partners (Figure 1).

## 3 Soil microbes for sustainable agriculture

### 3.1 Importance of soil microbes for plant health

**Figure 2:**
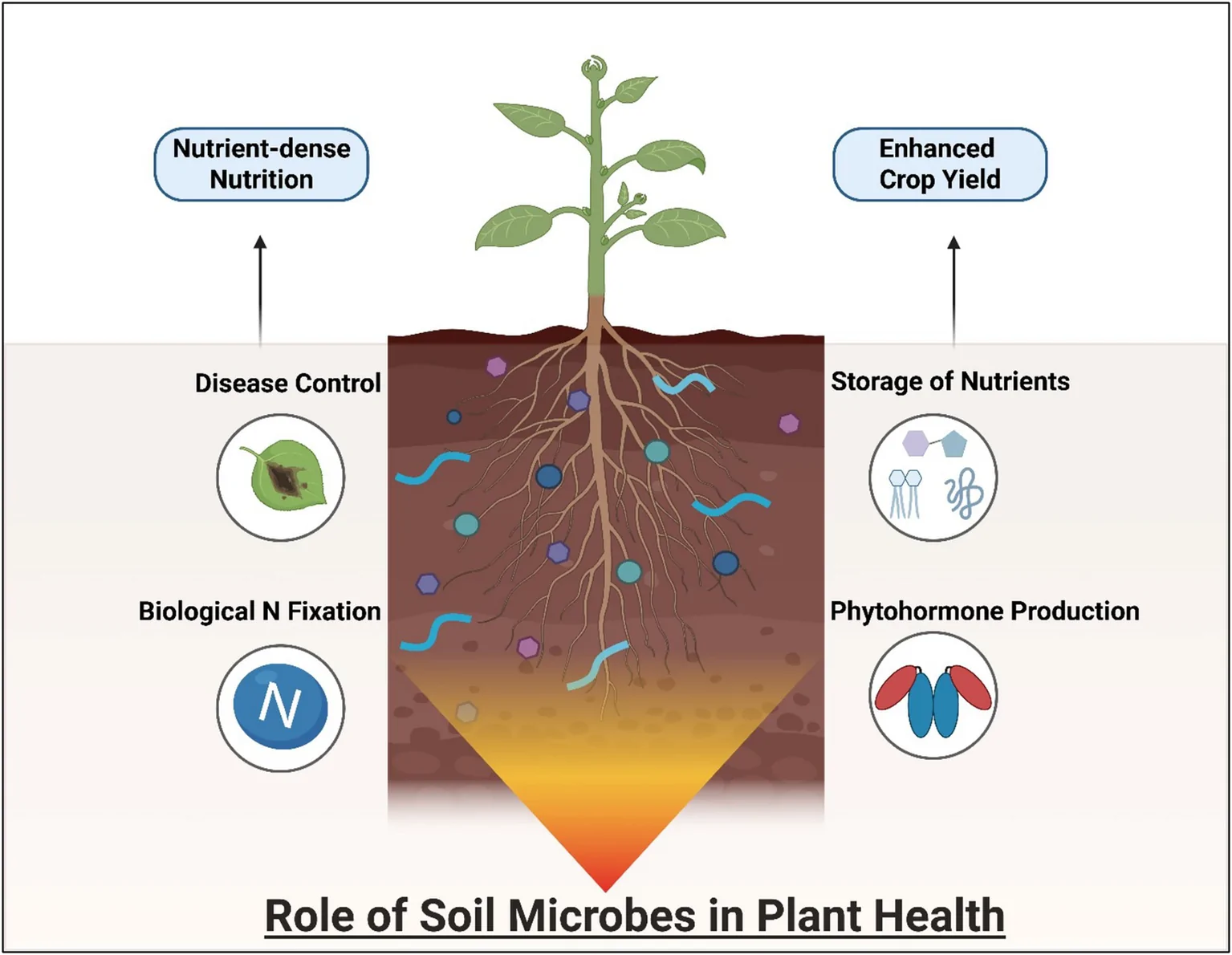
Advantages of soil microbes to improve plants’ health for sustainable agricultural production.

Soil forms the foundation of agricultural productivity, and microorganisms play a crucial role in shaping the complex interactions between plants and their environment. Microbial activity is therefore essential for restoring soil health and promoting sustainable crop growth. As a dynamic component of soil, microorganisms perform a wide range of beneficial functions within the soil system. They enhance nutrient availability, making it easier for plants to absorb essential elements (Figure 2). Despite their importance, microorganisms are often perceived negatively because of their association with diseases; however, from an agricultural perspective, they are highly beneficial for plant growth (Paries and Gutjahr, 2023).

Soil microorganisms facilitate interactions between soil and plant roots, recycle nutrients, decompose organic matter, and act as sensitive indicators of soil functional processes. They can also respond rapidly to changes in soil conditions (Jacoby et al., 2017). Globally, both symbiotic and non-symbiotic microorganisms are increasingly being utilized to enhance plant productivity (Ansari et al., 2024). Soil organic matter contains microbial biomass – the living components of organic matter – which plays a key role in nutrient cycling, transformation, and storage (Raza et al., 2023). The addition of organic matter stimulates both fungal and bacterial activity, thereby improving soil fertility and crop productivity (Shen et al., 2023).

Many soil microorganisms are beneficial to both soil health and plant growth (Thomopoulos et al., 2023). Beneficial microorganisms can suppress phytopathogens through various mechanisms, including competition for nutrients and space, thereby limiting pathogen growth (El-Saadony et al., 2022). They also promote plant development, enhance mineral uptake, and accelerate the decomposition of organic matter, leading to improved nutrient availability in soil (Li et al., 2023).

### 3.2 Role of microbial communication and signaling in plant health and productivity

Plants and microorganisms have coexisted for millions of years, forming complex and dynamic interactions that are essential for plant growth, nutrient acquisition, and defense responses. Communication between plants and their rhizospheric microbial communities occurs through a variety of signaling molecules that regulate physiological and biochemical processes. The exchange of signals between roots, shoots, and microorganisms is influenced by both biotic and abiotic factors and plays a critical role in plant development. Microorganisms produce a wide range of volatile and non-volatile compounds, including phytohormones, which can directly or indirectly regulate plant morphogenesis, growth, and immunity (Sablowski and Gutierrez, 2022).

**Figure 3:**
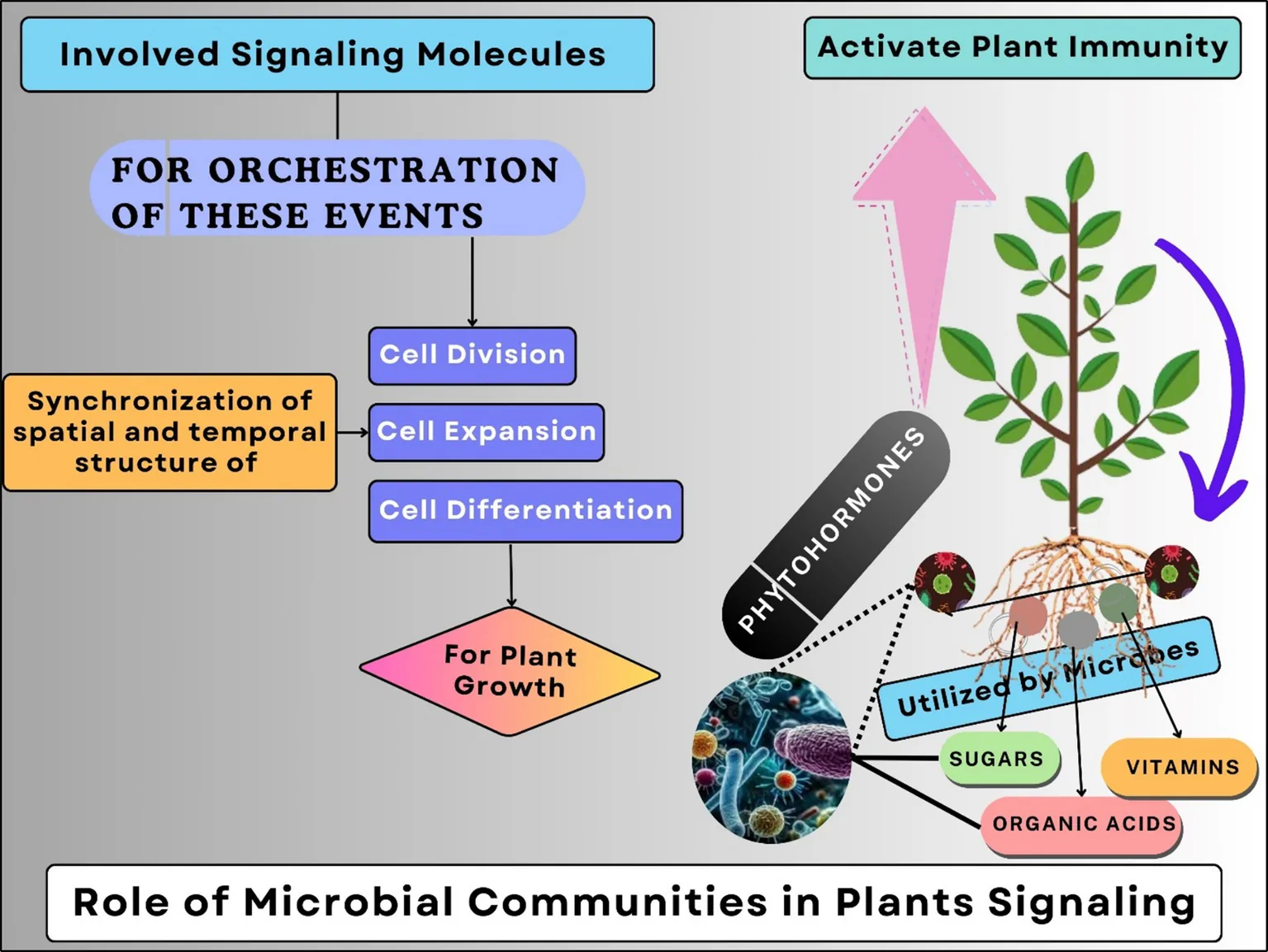
Role of soil microbial communities in plant signaling and immunity.

Throughout different stages of plant growth, signaling molecules produced by both plants and microorganisms facilitate communication and coordination (Figure 3). In the rhizosphere, fungi and bacteria recognize their plant hosts and initiate colonization through the production of signaling compounds, including auxins and cytokinins. In turn, plants perceive microbe-derived signals and adjust their growth and defense responses according to the nature of the interacting microorganisms. While considerable attention has been given to beneficial plant-microbe interactions that enhance plant productivity, recent studies have increasingly focused on identifying the signaling pathways involved in plant-pathogen communication (Mehta et al., 2021).

Improving rhizosphere management represents a promising biotechnological strategy for enhancing crop productivity while reducing dependence on water, fertilizers and agrochemicals. Such improvements might be achieved through the manipulation of root exudate composition or the introduction of beneficial microorganisms into the rhizosphere (Fazeli-Nasab et al., 2022).

Plant-microbe interactions are initiated through the exchange of specific signaling molecules produced by one or both partners during host recognition. This communication triggers a cascade of physiological, biochemical, and molecular responses that influence the development and adaptation of both plants and microorganisms (Pande et al., 2021). Consequently, the rhizosphere environment shaped by these interactions significantly affects root health, nutrient acquisition and overall plant performance (Khan et al., 2021).

### 3.3 Nutrient cycling and soil fertility enhancement by microbes

Microorganisms constitute one of the most abundant and diverse forms of life in soil and collectively form the soil microbiome. Soil microbial communities include bacteria, fungi, archaea, and other microscopic organisms that inhabit a wide range of soil types and environmental conditions (Mishra et al., 2016; Biswas et al., 2018). These microbes are indispensable for maintaining soil functions and supporting agricultural productivity. Soil microorganisms play a crucial role in nutrient cycling and organic matter decomposition. They regulate the transformation and availability of essential nutrients through biogeochemical cycles involving carbon, nitrogen, phosphorus, sulfur, and other minerals. The efficiency of these processes is strongly influenced by soil physicochemical properties, including pH, redox potential, organic matter content, and microbial biomass (Pandey et al., 2020). These factors also shape soil structure, ecosystem functioning, and microbial community composition (Vogel et al., 2024).

A key contribution of soil microorganisms to soil fertility is their ability to enhance nutrient availability. N-fixing microorganisms convert atmospheric N into plant-available forms, while phosphate- and potassium-solubilizing microorganisms transform insoluble nutrient pools into forms readily absorbed by plants. In addition, microbial decomposition of organic residues releases nutrients and contributes to the formation of stable soil organic matter, thereby improving soil fertility and sustainability.

Soil health is defined as the ability of soil to function as a living ecosystem that sustains plant productivity and environmental quality. Microorganisms are fundamental to this process because of their active involvement in nutrient cycling, organic matter turnover, and soil structure maintenance. The extensive functional and genetic diversity of microbial communities underpins their crucial role in maintaining soil fertility and supporting sustainable agricultural systems (Tahat et al., 2020; Qiao et al., 2024).

### 3.4 Plant growth promotion and disease suppression mechanisms

Plant growth-promoting microorganisms (PGPMs) enhance plant performance through a variety of direct and indirect mechanisms. Direct mechanisms include phytohormone production, nutrient mobilization, siderophore secretion, and improved nutrient acquisition. Through these activities, rhizosphere microorganisms stimulate seed germination, root development, nutrient uptake, and overall plant growth (Li et al., 2016).

Microorganisms also facilitate plant nutrition by increasing the availability of essential nutrients. Nitrogen-fixing bacteria convert atmospheric nitrogen into forms accessible to plants, while phosphate- and potassium-solubilizing microorganisms enhance the uptake of mineral nutrients. The abundance and activity of these beneficial microbes are influenced by factors such as soil type, fertility status, organic matter content, and management practices (Shao et al., 2016).

In addition to promoting plant growth, beneficial microorganisms contribute significantly to disease suppression. Many bacterial and fungal species produce antibiotics, lytic enzymes, siderophores, and other bioactive compounds that inhibit the growth and proliferation of pathogens (Huang et al., 2017). In addition, beneficial microbes compete with pathogens for nutrients and ecological niches, reducing pathogen establishment and infection. Plants possess a sophisticated neutral defense system composed of three primary components: the plant immune system, endophytes, and the rhizosphere microbiome (Enebe and Babalola, 2019). Together, these components enhance plant resilience against pathogen attack. Beneficial microorganisms can also induce systemic resistance, thereby enhancing host defense responses and improving disease tolerance. Activating synergistic interactions among these defense layers represents a sustainable strategy for disease management (Xiong et al., 2017).

Although pathogens are present in most soils, some soils exhibit natural disease suppressiveness. These disease-suppressive soils support healthy plant growth despite the presence of phytopathogens (Nwokolo et al., 2021). In such environments, disease development is limited by beneficial microbial communities that suppress pathogen activity and enhance plant defense responses (Singh et al., 2023). Appropriate soil management practices that enhance beneficial microbial activity can therefore contribute to the development of disease-suppressive soils and improve crop productivity.

The synergistic application of beneficial microorganisms and organic amendments has been widely reported as an effective strategy for plant growth promotion and disease control (Fu et al., 2017). For example, Bacillus species produced through solid-state fermentation on manure-based substrates have demonstrated significant effectiveness in controlling Fusarium wilt in banana plants (Yadav et al., 2023). However, many microbial formulations remain region-specific, and their broader applicability requires further validation under diverse agricultural conditions.

### 3.5 Impact of microbial diversity on plant productivity

PGPMs are a diverse group of beneficial microorganisms that directly or indirectly enhance plant growth and productivity (Abhilash et al., 2016). These microbes play a critical role in sustainable agriculture by improving soil fertility, increasing crop productivity, promoting microbial diversity and beneficial interactions, suppressing phytopathogens, and maintaining long-term agroecosystem stability (Singh et al., 2020). Microbial communities contribute significantly to the maintenance of nutrient-rich and biologically active soils. Biological fertilization mediated by these microbes has been widely recognized as an effective strategy for supplying essential nutrients to plants. The growth-promoting effects of soil microbes are generally explained by three primary mechanisms: modulation of plant hormonal signaling, suppression or competition with pathogenic microbes, and enhancement of mineral nutrient availability (Bender et al., 2016; Verbon and Liberman, 2016). Soil microorganisms produce a wide range of phytohormones, including auxins, gibberellins, salicylic acid (SA), abscisic acid (ABA), and cytokinin, all of which play important roles in regulating plant growth and development. These microbially derived hormones are increasingly recognized as potential targets for metabolic and biotechnological interventions aimed at improving plant performance under abiotic stress conditions (Egamberdieva et al., 2017).

The plant root system serves as a dynamic biochemical interface that mediates interactions between plants and soil microorganisms. Root exudates, composed of various organic compounds, act as signaling signals that attract beneficial microbes while inhibiting harmful ones. These exudates not only mediate plant-microbe interactions but also significantly influence the diversity, abundance, and activity of microbial communities in the rhizosphere. Moreover, root exudation patterns are shaped by both biotic and abiotic environmental factors. Plant-associated microorganisms enhance plant growth and health through multiple complementary mechanisms, including biological nitrogen fixation, production of vitamins and phytohormones, improved nutrient acquisition, and enhanced tolerance to environmental stresses (Qiao et al., 2024). Overall, these interactions between microbial diversity and plant systems play a crucial role in improving crop productivity and sustaining agricultural ecosystems.

## 4 Factors affecting plant and soil microbe interactions

Plants and soil microorganisms interact through the exchange of resources and the modification of microhabitat conditions. Variations in soil resources directly influence the composition and structure of microbial communities (Philippot et al., 2024). For example, changes in the soil carbon-to-nitrogen (C) ratio can alter the composition of bacterial and fungal communities. The availability and quality of substrates also strongly affect microbial decomposition processes because different microbial groups vary in biomass composition and substrate-use efficiency (Raza et al., 2023).

Many organisms can modify their immediate environment, thereby influencing their own performance and that of neighboring organisms. Plants, for instance, alter soil conditions, which subsequently affect the growth and development of both themselves and surrounding plants. These changes are often associated with plant–soil feedback mechanisms, in which soil microbial communities play a central role (Frouz, 2023). The accumulation of pathogens can suppress plant growth, whereas the enrichment of beneficial microorganisms, such as mycorrhizal fungi and nitrogen-fixing bacteria, can enhance plant performance. Plants release a variety of bioactive compounds into the rhizosphere that shape the soil microbiota. Root exudates commonly contain primary metabolites, including sugars, amino acids, and carboxylic acids, as well as numerous secondary metabolites (Ma et al., 2022). In addition to serving as carbon and nitrogen sources for microbial growth, these compounds influence microbial activity, abundance, and community composition in the rhizosphere (Baetz and Martinoia, 2014).

Secondary metabolites are also involved in the interactions among endophytes, phytopathogens, and host plants. Endophytic fungi are known to produce a wide range of biologically active secondary metabolites that contribute to plant growth promotion and disease resistance (Prakash, 2015). These interactions play important ecological roles, including enhancing plant fitness and protecting plants against phytopathogenic microorganisms (Mishra et al., 2021).

Differences in the physiology and growth rates of soil organisms can alter the abundance and functional roles of microbial communities in response to climate change (Briones et al., 2014). For example, a 5 °C increase in temperature in a temperate forest ecosystem was shown to modify the bacterial-to-fungal ratio and alter the relative abundance of soil bacterial populations (DeAngelis et al., 2015). Changes in microbial community composition can affect ecosystem functions when microorganisms regulate key processes or possess distinct functional traits (Li et al., 2024; Zhao et al., 2025). Certain microbial groups are responsible for critical processes such as nitrification, nitrogen fixation, denitrification, and methanogenesis, all of which are essential for ecosystem functioning (Monib et al., 2024). Consequently, changes in the abundance of these microorganisms can directly influence the rates of these processes. However, broader ecosystem functions, such as nitrogen mineralization, are often more strongly influenced by abiotic factors, including temperature and soil moisture, than by microbial community composition alone (Sokol et al., 2022).

Various ecological factors can also drive shifts in soil microbial communities. Plant-related factors, including productivity gradients and plant–soil feedbacks, have been shown to influence both microbial community composition and ecosystem processes (Sveen et al., 2024). While most studies have focused on differences among plant species or functional groups, increasing evidence suggests that the genotypes of individual plants can also influence associated belowground microbial communities, and vice versa. Plant litter inputs, both aboveground and belowground, affect substrate availability for soil microorganisms and can regulate microbial community structure (Zhang et al., 2023).

Rhizosphere microbial communities are further shaped by the interaction between plant genetics and fertilization practices (Abubakar et al., 2023). The rhizosphere, a biologically active zone at the interface between plant roots and soil, plays a critical role in nutrient cycling, ecosystem functioning, and plant health (Benaissa, 2024). Plant roots release exudates that stimulate microbial activity and influence microbial community structure, while microorganisms provide nutrients and other benefits that support plant growth (Paterson et al., 2007). Root exudates contain a diverse range of compounds, including sugars, amino acids, enzymes, and siderophores. The composition of these exudates is influenced by factors such as plant species, genotype, developmental stage, and fertilization regime. Consequently, variations in root exudation patterns affect both the composition of the soil microbial pool and the structure of rhizosphere microbial communities (Anderson et al., 2024).

## 5 Strategies for enhancing beneficial microbe-plant interactions

Beneficial plant-microbe interactions play a crucial role in improving plant growth, soil health, nutrient use efficiency, and disease management (Table 2). These mutualistic relationships form the foundation of sustainable agriculture, in which plants provide carbon-rich substrates to rhizospheric microorganisms, which, in turn, enhance nutrient acquisition, stress tolerance, and plant productivity. Therefore, developing strategies to strengthen these positive interactions is essential for improving plant health, productivity, and resilience.

**Table 2:** Factors influencing plant–soil microbes interactions.

Factors	Description	Influence on interactions	References
Atmospheric CO_2_	Influenced the distribution and composition of carbon in the substances released by plant roots	Altered plant physiology and metabolism. Reshaped the root exudate composition.	Compant et al. (2010)
UV-radiations	Greater exposure of plants to radiation, which in turn altered ozone dynamics in the atmosphere.	Changed cell oxygen yield, affected the efficacy of the photosynthetic system growth of cyanobacteria	Zeeshan and Prasad (2009)
Soil pH	The pH of soil has a crucial role in determining the solubility of various metal ions, the availability of nutrients, and the physical qualities of the soil.	Too high or too low pH affects plant productivity by causing nutrient deficiency	Msimbira and Smith (2020)
Water	Precipitation is the primary water source for crop cultivation in arid areas. Waterlogged or drought conditions are major yield-limiting factors.	Drought inhibits microbial communities. Flooding reduces oxygen availability and, in turn, potash assimilation	Danish et al. (2020)
Light	Influenced plant metabolism and growth. Controlled the amount and interaction of root exudates.	Low light inhibits microbial growth and interactions. Also, reduces photosynthesis and carbohydrate production	Ballhorn et al. (2016)
Temperature	Optimum temperature is crucial for plant growth and metabolism, and soil microbial associations.	Impacted morphological, biochemical, and physiological mechanisms. Interfere with PGPM-plant interactions and change the root exudate composition.	İpek et al. (2019).

### 5.1 Soil microbial inoculants

Microbial inoculants are formulations containing beneficial bacteria, fungi, and other microorganisms that are introduced into agricultural systems to enhance plant growth, nutrient availability, and disease suppression (Kaminsky et al., 2019). These inoculants have emerged as environmentally friendly alternatives to chemical fertilizers and pesticides, contributing to sustainable crop production.

Beneficial microbial groups used as inoculants include PGPR, mycorrhizal fungi, and actinomycetes. PGPR colonize plant roots and improve plant performance through mechanisms such as biological N fixation, phosphate solubilization, phytohormone production, and induction of systemic resistance. Common PGPR genera include *Pseudomonas*, *Serratia*, *Azoarcus*, *Azospirillum*, *Rhizobium*, *Azotobacter*, *Clostridium*, *Enterobacter*, and Gluconacetobacter (O’Callaghan et al., 2022). Mycorrhizal fungi enhance nutrient uptake, particularly phosphorus acquisition, while also contributing to soil aggregation through the production of glomalin and extensive hyphal networks (Prasad, 2021). Actinomycetes contribute to organic matter turnover, disease suppression, and plant growth promotion, further supporting soil health and productivity (O’Callaghan et al., 2022). In addition, certain soil bacteria produce biosurfactants that facilitate root colonization, biofilm formation, and activation of plant defense responses (Silva et al., 2024).

Seed and soil inoculation with beneficial microbes has been shown to improve root establishment, nutrient uptake, and resistance to biotic and abiotic stresses (Ahmad et al., 2018). The use of microbial consortia, consisting of multiple compatible microbial species, is particularly effective because of their ability to establish stable associations within the rhizosphere and provide complementary plant-beneficial functions. Furthermore, these microorganisms improve nutrient cycling, enhance soil fertility, and increase carbon sequestration (Zhao et al., 2023).

Nitrogen deficiency is a major constraint to agricultural productivity. Therefore, nitrogen-fixing bacteria that form symbiotic associations with legumes remain central to biofertilizer development. In soil with low native rhizobial populations, inoculation with appropriate strains is often essential to maximize biological nitrogen fixation and crop productivity (Wu and Qiu, 2024).

### 5.2 Crop rotation and cover crops

Cover crops (CCs) provide multiple ecosystem services, including reducing soil erosion, enhancing carbon sequestration, improving water infiltration, reducing nutrient leaching, and suppressing pests and weeds (Adetunji et al., 2020; Sharma et al., 2018). Cover cropping is the practice of integrating these crops into the farming systems (Aglasan et al., 2024). Cover crops enhance plant and microbial diversity and regulate biogeochemical nutrient cycling, thereby reducing reliance on synthetic fertilizers (Kuhlisch et al., 2024). They also improve soil biological fertility by supporting earthworms and microbial communities (Vukicevich et al., 2016). Additionally, root architecture improves soil aggregation, porosity, and moisture retention (Haruna et al., 2020).

Legumes play a particularly important role in soil fertility improvement due to their ability to fix atmospheric nitrogen through symbiosis with microorganisms (Vasconcelos et al., 2020). Their residues, especially those with low carbon-nitrogen ratios, increase nitrogen availability and reduce fertilizer requirements (Adetunji et al., 2020). Integrating legume and non-legume cover crops optimizes nitrogen cycling and reduces nitrogen losses (Latati et al., 2019).

### 5.3 Compost and organic amendments

Soil organic matter is a primary indicator of soil quality and is essential for the long-term sustainability of agroecosystems and global biogeochemical cycles (Rastogi et al., 2023). Organic amendments such as composts and animal manures have long been used in agriculture due to their ability to supply nutrients and improve plant health. However, reliance on agrochemicals has reduced their widespread use (Iyiola et al., 2023). The quality and quantity of organic matter added to soil vary depending on management practices, and these inputs significantly influence both the biological components and physicochemical characteristics of soil, thereby affecting the abundance, diversity, and functions of soil microbiota (Daunoras et al., 2024).

Application of compost and other organic amendments increases microbial biomass compared to untreated or chemically fertilized soils (Rombel et al., 2022; Yuan et al., 2026) and enhances microbial diversity (Wang et al., 2024). These amendments also increase soil microbial activity, reflected in higher soil respiration rates and enzymatic activity such as fluorescein diacetate (FDA) hydrolysis (Ye et al., 2024). In addition, organic amendments improve plant health by suppressing plant pathogens, including nematodes, bacteria, and fungi (Naghman et al., 2023).

### 5.4 Biological pest control

Biological control is an effective approach for managing plant diseases (Etesami et al., 2023). It involves the use of living organisms, other than resistant host plants, to suppress the growth, activity, or reproduction of plant pathogens. Disease-suppressive soils harbor diverse antagonistic microorganisms. Common agents include fungi such as *Penicillium notatum*, *Sporidesmium*, and *Trichoderma harzianum*, as well as bacteria such as *Pseudomonas Putida*, *Bacillus subtilis*, and *Streptomyces coelicolor*. When conductive soils are amended with suppressive soils, beneficial microorganisms are introduced, reducing disease incidence (Agrios, 2005).

Biopesticides are pest control agents produced from natural substances or living microorganisms and are classified into microbial agents (bacteria, fungi, viruses, and oomycetes) and biochemical agents (plant-derived compounds such as essential oils, chitosan, and chitin). Compared with chemical pesticides, biopesticides are more environmentally sustainable, less toxic, and exhibit diverse modes of action. However, excessive reliance on chemical pesticides can lead to environmental contamination, pathogen resistance, and harmful residues in food (Singh et al., 2024).

### 5.5 Microbial consortia

Microbial consortia consist of multiple compatible microbial species that functionally synergistically, often outperforming single strains in agricultural application (Wanapaisan et al., 2018). Single microbial strains are frequently insufficient for the complete degradation of complex pollutants due to limited metabolic capacity. In contrast, microbial consortia utilize complementary metabolic pathways, resulting in enhanced pollutant degradation efficiency (Varjani et al., 2021).

Single-strain inoculants often fail under field conditions due to poor colonization and environmental variability (Qiu et al., 2019). Synthetic microbial consortia address these limitations by combining multiple functional strains into a stable community. These consortia enhance nutrient cycling, plant growth promotion, disease suppression, and bioremediation of contaminated environments, making them a promising strategy for sustainable agriculture (Kaur et al., 2022).

### 5.6 Biofertilizers

Biofertilizers are microbial formulations that enhance plant growth by increasing nutrient availability through colonization of the rhizosphere or plant tissues. They can be applied to seeds, plant surfaces, or soil and represent a sustainable alternative to synthetic fertilizers. These inoculants have been reported to increase yield by 10–40% (Song et al., 2023). They improve nutrient uptake, enhance root development, increase seedling survival, and improve plant tolerance to environmental stresses (Sivaram et al., 2023). Once established, biofertilizers may persist in soil, reducing the need for repeated applications (Bumandalai and Tserennadmid, 2019). Common biofertilizers include nitrogen-fixing bacteria, phosphate-solubilizing microbes, cyanobacteria, and other beneficial microbes that contribute to sustainable nutrient management (Umesha et al., 2018).

### 5.7 Precision agriculture techniques

Precision agriculture is a data-driven farming approach that integrates advanced technologies to optimize resource use, improve crop productivity, and enhance agricultural sustainability (Kumar et al., 2020). Hydroponics involves growing plants in nutrient-rich water, enabling precise control of nutrients and environmental conditions, resulting in faster plant growth and improved crop quality (Tambakhe and Gulhane, 2020). Aquaponics integrates aquaculture and hydroponics, where fish waste is converted into plant-available nutrients by nitrifying bacteria (Diver and Rinehart, 2000). Aeroponics involves growing plants with roots suspended in air and periodically misted with nutrient solutions, enabling high productivity with minimal water use (Lakhiar et al., 2020).

Precision agriculture also uses technologies such as GPS and GIS for field mapping, special data analysis, and input optimization (Zhang and Cao, 2019). These tools enable site-specific management of water, fertilizers, and pesticides, improving efficiency and sustainability. Additionally, modern sensor-based irrigation systems enhance water-use efficiency, particularly in water-limited regions (García et al., 2020).

## 6 Techniques for studying plant-microbe interactions

### 6.1 Omics techniques

#### 6.1.1 DNA sequencing

Recently, genomic analysis of environmental samples has become one of the most effective approaches for understanding evolutionary history, structure, function, and ecological diversity (Fazeli-Nasab et al., 2022). Metagenomics is the most widely used technique to investigate complex microbial communities in environmental samples by analyzing nucleotide sequences (Mukherjee et al., 2023). Metagenomic studies primarily employ two techniques: targeted (amplicon) sequencing and shotgun sequencing. The choice between these strategies depends on the objectives of the environmental study.

Next,-generation sequencing (NGS) technologies enable direct extraction and analysis of DNA sequences from environmental samples. Among available platforms, Illumina sequencing is most commonly used due to its high accuracy, high throughput, and cost-effectiveness, generating millions of short reads (Pervez et al., 2022). Various techniques used for studying microbial communities are summarized in Table 3.

**Table 3:** Approaches for studying plant–soil microbes interactions.

Approaches	Description	Applications	Reference
Microscopy	This method is highly beneficial for examining the surface of various microorganisms and identifying the ecological niches they inhabit, as well as understanding the cytotoxic pathogenic properties of bacteria.	SEM, TEM and light microscopy are employed to examine the surface of various microorganisms and identify their ecological niches. These techniques also provide insights into the cytotoxic and pathogenic capacities of bacteria.	Chen et al. (2011)
Nucleic acid extraction	Used to identify genes in the soil microbial community that yield dependable and expeditious outcomes. This method is highly favored by the scientific community.	Nucleic acids are extracted using three distinct techniques, which are determined by the nature of the sample and the intended use of the recovered material. (a) Fragmenting the samples (tissue or cells); (b) Purifying the nucleic acid by eliminating lipids, proteins, and other impurities; (c) Transferring the nucleic acid to a buffer solution for preservation.	Sirakov (2016)
Nucleic acid hybridization	Nucleic acid hybridization, specifically DNA–DNA and RNA–DNA hybridization, is a valuable approach in the field of plant microbiome research. This approach involves the quantitative analysis of nucleic acid using particular probes.	Fluorescence in situ hybridization used in molecular biology to detect and locate specific DNA sequences within cells or tissues. This method can be employed to examine the spatial distribution of a microbiological population in a distinct location.	Ratan et al. (2017)
Polymerase chain reaction	PCR is a rapid, user-friendly, highly sensitive, reproducible, and universally applicable method. The approach is cost-effective, efficient, utilizes minimal resources, and has a high processing capacity.	The RT-PCR enzyme measures the extent of gene impacts in certain environments and the level of gene activity in small habitats.	Garibyan and Avashia (2013)
Transcriptomics	The primary objective of transcriptomic analysis of plant-associated bacteria utilizing RNA sequencing (RNA-seq) or gene expression microarray techniques is to identify genes that exhibit differential expression under various environmental circumstances.	Used to identify bacterial genes that are expressed differently throughout the growth of Arabidopsis and invasion by a fungal pathogen.	Coutinho et al. (2015)
Nucleic acid amplification and fingerprinting	Polymerase chain reaction (PCR) is used to amplify DNA, whereas Complementary DNA (cDNA) is synthesized from the isolated RNA. The amplicons can be analyzed utilizing fingerprinting techniques such as denaturing gradient gel electrophoresis, terminal restriction fragment analysis, amplified rDNA restriction analysis, and BOX-PCR.	Bioactive compound production-Essential oils and Remediation-Toluene and BTEX	Wu et al. (2009)

#### 6.1.2 Sequencing of endophytic genes

A wide range of agriculturally significant microorganisms reside within plant internal tissues (Gupta et al., 2021; Adeleke et al., 2022). Endophytic bacteria play diverse roles in agriculture, including enhancing plant growth and mitigating environmental stresses (Mahgoub et al., 2021). Their ability to support plant growth under stress is linked to the production of volatile and bioactive substances, as well as enzymes such as ACC deaminase, which lowers stress-induced ethylene levels. Plant tissue colonization by microbes generally occurs in five stages: (i) molecular communication between plant and bacteria through root exudates and chemotaxis; (ii) attachment to the root surface; (iii) biofilm formation; (iv) penetration into the root cortex; and (v) internal colonization (Kandel et al., 2017). These processes are regulated by dynamic gene expression changes in both the host plant and microbes. Multiple-omics approaches are therefore essential to better understand the molecular mechanisms underlying these interactions.

#### 6.1.3 Metabolomics

**Figure 4:**
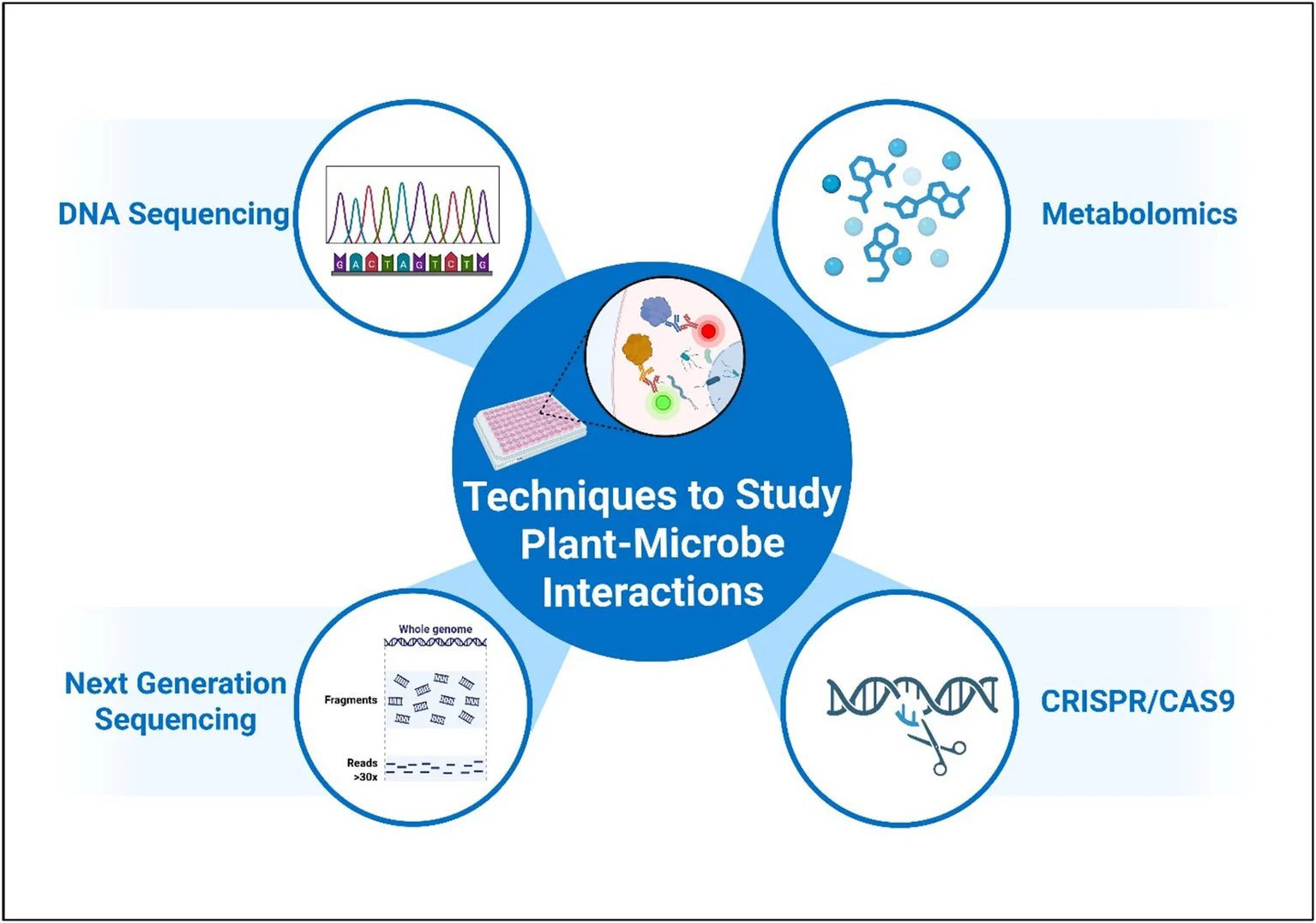
The commonly used techniques to study plant-microbe interactions.

Metabolomics focuses on the comprehensive analysis of metabolites and their dynamic changes in response to environmental conditions (Figure 4). The fundamental components of any metabolomics approach are metabolite detection, identification, and quantification. Plants host a diverse microbiota, including actinomycetes, bacteria, fungi, algae, and protozoa (Pirzadah et al., 2021). Among these, bacteria are the most dominant group. PGPB colonize the rhizosphere and contribute significantly to plant growth and stress tolerance. Fungi such as Aspergillus, Penicillium, and Trichoderma also play important roles in nutrient cycling and organic matter decomposition in agricultural and natural ecosystems (Zeilinger et al., 2016). Plant-fungal interactions are highly complex and may range from mutualism to parasitism depending on environmental conditions.

#### 6.1.4 Next-generation sequencing

Next-generation sequencing (NGS) technologies have significantly advanced biological research by enabling rapid and cost-effective generation of large-scale sequence data. These technologies allow comprehensive analysis of microbial communities associated with plants, providing insights into their structure, function, and diversity. Metagenomic and genomic approaches using NGS help elucidate the physiological capabilities of microbial populations. When combined with RNA-based analyses (metatranscriptomics), they further reveal active metabolic pathways and regulatory mechanisms under specific environmental conditions (Wang et al., 2026). Overall, NGS has greatly expanded the ability to study plant–microbe interactions by overcoming previous technical and financial limitations, enabling faster and more detailed investigations than ever before.

#### 6.1.5 CRISPR-based study of plant-microbe interactions

Genetic techniques based on CRISPR have transformed our capability to examine and modify genes. By revealing their genetic basis, these technologies may be used for both host and microbial cells, improving our comprehension of the dynamic nature of plant-microbe interactions. The microbiome is being investigated in a high-throughput, accurate, and efficient manner using CRISPR-based approaches. Through the use of recording devices, targeted genome editing, and CRISPR screens, host cells and microbes are capable of being studied (Echavarria Galindo and Lai, 2025).

## 7 Limitations and prospects

### 7.1 Limited understanding of complex microbial communities

Microorganisms inhabit nearly every environment on Earth, forming diverse and highly complex ecosystems. Interactions among microbial communities create intricate and interconnected networks that are shaped by both biotic and abiotic factors. In natural ecosystems, microbial diversity is closely associated with a wide range of metabolic functions involved in essential biogeochemical processes. Furthermore, microorganisms interact directly with higher organisms, including plants, influencing ecosystem functioning and productivity (Guidi et al., 2016; Louca et al., 2016).

Plant-microbe interactions are regulated by a variety of signaling mechanisms; however, only a limited number of these signals have been thoroughly studied. This knowledge gap is reflected in the well-known observation attributed to Leonardo da Vinci, who suggested that our understanding of celestial bodies exceeds our knowledge of the processes occurring beneath our feet (Badri et al., 2009). Although numerous traditional and advanced techniques have been used to explore the structure, abundance, and function of soil microorganisms, these approaches provide only partial insights into the complexity of microbial communities. While they contribute valuable information, they do not fully explain the dynamic interactions occurring between plants and microorganisms under diverse environmental conditions. Therefore, more integrated and multidisciplinary approaches are required to comprehensively elucidate the mechanisms governing plant-microbe interactions in natural and agricultural ecosystems.

### 7.2 Lack of standardized methodologies

Despite significant advances in methods for identifying and characterizing soil microorganisms, accurately quantifying microbial community structure and function remains a major challenge. Microbial interactions are highly complex and involve numerous signaling molecules and communication mechanisms that enable specific microbes to recognize and respond to one another within dynamic environments characterized by fluctuating physicochemical conditions and diverse host influences. Although several of these mechanisms have been discussed in this review, much remains unknown regarding the signaling compounds, biomarkers, and molecular communication processes involved in plant-microbe interactions.

To achieve a deeper and more accurate understanding of these interactions, further investigations at the molecular level are essential. There is a critical need for the development and standardization of advanced analytical tools, methodologies, and both *in vitro* and *in vivo* experimental models. One of the major challenges lies in integrating genomic information, gene expression profiles, and metabolite dynamics within complex microbial communities and environmental contexts.

Microorganisms influence their hosts through both direct and indirect mechanisms. The advent of next-generation sequencing technologies has greatly enhanced our ability to explore these interactions at the molecular level. Omics approaches – such as genomics, transcriptomics, proteomics, and metabolomics – have generated extensive datasets that have improved our understanding of plant-microbe interactions, and their role in plant growth and development, immune responses, nutrient cycling, nutrient acquisition, and biological pest control. Most studies to date have focused on identifying dominant microbial taxa within specific environments, investigating how plant-derived compounds (e.g., root exudates) influence microbial recruitment, and characterizing community structure and diversity. However, further research is needed to elucidate interactions among microbial communities, the mechanisms underlying microbial recruitment, and the factors governing plant-microbe associations. In addition, the effects of microbial communities on root architecture and exudation patterns remain poorly understood. A more comprehensive understanding of these processes will facilitate the strategic manipulation of microbial populations to improve plant performance and promote sustainable agricultural systems.

## 8 Conclusion

Recent advances in genome-based research technologies have enabled more precise characterization of the diversity, genomes, and proteomes of microorganisms inhabiting soil and plant environments. Omics-based approaches, including genomics, transcriptomics, proteomics, metagenomics and DNA- and RNA-based analyses, have revolutionized the study of plant-microbe interactions by providing unprecedented depth and resolution compared with traditional methods. These technologies facilitate a comprehensive understanding of both above-ground and belowground biological processes and enable the application of this knowledge to improve plant growth, crop productivity, and disease resistance. Soil microorganisms play essential roles in key ecological processes, including nutrient recycling, decomposition, organic matter turnover, and nutrient transformation. Active microbial communities are particularly important for regulating nutrient release from organic matter and ensuring nutrient availability to plants, especially in organic and low-input agricultural systems. In addition, beneficial soil microorganisms contribute to disease suppression through mechanisms such as antibiosis, competition for nutrients and ecological niches, and the induction of plant defense responses.

A better understanding of the factors that shape microbial community assembly and function within the rhizosphere will support the development of more sustainable, productive, and resilient agroecosystems. Such knowledge can facilitate ecosystem restoration, improve resource use efficiency, and maintain long-term agricultural productivity. Furthermore, advances in omics technologies and the expanding availability of genomic datasets for plants, microorganisms, and phytopathogens have significantly enhanced our capacity to investigate plant-microbe interactions at multiple biological scales. Continued integration of these technologies with ecological and agronomic research will be essential for harnessing beneficial microbial communities and advancing sustainable agricultural practices in the future.
